# Effect of Environmental Tobacco Smoke on Levels of Urinary Hormone Markers

**DOI:** 10.1289/ehp.7436

**Published:** 2005-01-14

**Authors:** Changzhong Chen, Xiaobin Wang, Lihua Wang, Fan Yang, Genfu Tang, Houxun Xing, Louise Ryan, Bill Lasley, James W. Overstreet, Joseph B. Stanford, Xiping Xu

**Affiliations:** ^1^Department of Environmental Health, Harvard School of Public Health, Boston, Massachusetts, USA; ^2^Department of Pediatrics, Northwestern University Feinberg School of Medicine and Children’s Memorial Hospital and Children’s Memorial Research Center, Chicago, Illinois, USA; ^3^Center for Ecogenetics and Reproductive Health, Beijing Medical University, Beijing, China; ^4^Institute for Biomedicine, Anhui Medical University, Anhui, China; ^5^Department of Biostatistics, Harvard School of Public Health, Boston, Massachusetts, USA; ^6^Institute of Toxicology and Environmental Health and Department of Obstetrics and Gynecology, School of Medicine, University of California, Davis, California, USA; ^7^Health Research Center, Department of Family and Preventive Medicine, University of Utah, Salt Lake City, Utah, USA

**Keywords:** environmental tobacco smoke, estrone conjugates (E_1_C), pregnanediol-3-glucuronide (PdG), prospective study, urinary hormone levels

## Abstract

Our recent study showed a dose–response relationship between environmental tobacco smoke (ETS) and the risk of early pregnancy loss. Smoking is known to affect female reproductive hormones. We explored whether ETS affects reproductive hormone profiles as characterized by urinary pregnanediol-3-glucuronide (PdG) and estrone conjugate (E_1_C) levels. We prospectively studied 371 healthy newly married nonsmoking women in China who intended to conceive and had stopped contraception. Daily records of vaginal bleeding, active and passive cigarette smoking, and daily first-morning urine specimens were collected for up to 1 year or until a clinical pregnancy was achieved. We determined the day of ovulation for each menstrual cycle. The effects of ETS exposure on daily urinary PdG and E_1_C levels in a ±10 day window around the day of ovulation were analyzed for conception and nonconception cycles, respectively. Our analysis included 344 nonconception cycles and 329 conception cycles. In nonconception cycles, cycles with ETS exposure had significantly lower urinary E_1_C levels (β= –0.43, SE = 0.08, *p* < 0.001 in log scale) compared with the cycles without ETS exposure. There was no significant difference in urinary PdG levels in cycles having ETS exposure (β= –0.07, SE = 0.15, *p* = 0.637 in log scale) compared with no ETS exposure. Among conception cycles, there were no significant differences in E_1_C and PdG levels between ETS exposure and nonexposure. In conclusion, ETS exposure was associated with significantly lower urinary E_1_C levels among nonconception cycles, suggesting that the adverse reproductive effect of ETS may act partly through its antiestrogen effects.

In many regions of the world, tobacco use is much more common among men than among women. In China, for example, 63% of men and only 3.8% of women are estimated to be smokers (Yang et al. 1999). Nevertheless, research has consistently shown that many women are exposed to environmental tobacco smoke (ETS), based both on self-reports and on biologic indicators of exposure (Windham et al. 1999a). Thus, the effects of ETS on reproductive health are of major public health importance.

Many studies have shown that active smoking has adverse effects on a broad spectrum of reproductive outcomes, including fertility (Curtis et al. 1997; Howe et al. 1985; Hughes and Brennan 1996), time to pregnancy (Baird and Wilcox 1985; Bolumar et al. 1996; Curtis et al. 1997; Hull et al. 2000), spontaneous abortion (Chatenoud et al. 1998; Himmelberger et al. 1978; Kline et al. 1977), and birth weight (Lindbohm et al. 2002; Wang et al. 2002; Windham et al. 2000). Active smoking has also been associated with menstrual disturbances (Windham et al. 1999b). Some studies have suggested that smoking may reduce levels of estrogen and possibly progesterone, although results have been mixed (Basu et al. 1992; Friedman et al. 1987; Jensen and Christiansen 1988; Key et al. 1991; Khaw et al. 1988; Longcope and Johnston 1988; MacMahon et al. 1982; Michnovicz et al. 1986, 1988; Mochizuki et al. 1984; Slemenda et al. 1989; Thomas et al. 1993; Westhoff et al. 1996).

The evidence regarding adverse effects of ETS on reproductive outcomes is less robust. Studies have shown that ETS in the absence of maternal smoking significantly reduced infant birth weight (Martin and Bracken 1986; Rubin et al. 1986) and increased risk of preterm birth (Hanke et al. 1999; Jaakkola et al. 2001). Studies of the effects of ETS on spontaneous abortion have been inconsistent (Ahlborg and Bodin 1991; Chatenoud et al. 1998; Samet 1991; Windham et al. 1992, 1999c). Our studies have shown a significant dose–response relation between ETS exposure and dysmenorrhea (Chen et al. 2000) and early pregnancy loss (Venners et al. 2004).

In this study, we investigated the effect of ETS exposure on the levels of urinary pregnanediol-3-glucuronide (PdG; the major metabolite of progesterone) and estrone conjugates (E_1_C; the major metabolite of estrogen) in a cohort of women who participated in a reproductive health study in Anhui, China. These women were followed prospectively from the beginning of stopping contraception and attempting to conceive, through the end of pregnancy (or for up to 1 year without conception). The women provided daily diary records of vaginal bleeding and active and passive smoking and also submitted daily urinary specimens, which permitted us to accurately determine the onset of each menstrual cycle, the day of ovulation, and hormone levels specific to cycle day relative to ovulation. This was a homogeneous cohort of young women, all of whom were newly married, nulliparous, and employed full time in the textile industry. Few of the women we studied smoked cigarettes, but exposure to ETS was very high because of the high prevalence of smoking among their husbands. Few women used pills or hormone shots as contraceptive methods before entering the cohort. These characteristics provided a unique opportunity to study the adverse effects of ETS exposure on reproductive hormones while minimizing potential confounding factors.

## Materials and Methods

### Study population and procedures.

This is part of a large prospective reproductive health study among women textile workers in Anhui, China. The study protocols were approved by the human subject committees of the Chinese institutions involved in the study and by the institutional review board of the Harvard School of Public Health.

Detailed description of field data collection can be found elsewhere (Wang et al. 2003). Briefly, the eligibility criteria for the field enrollment were as follows: *a*) full-time employed women workers, *b*) newly married, *c*) 20–34 years of age, and *d*) had obtained permission to have a child. All the women were nulliparous. Women were excluded if *a*) they were already pregnant before enrollment, *b*) they had tried unsuccessfully to get pregnant for at least 1 year in the past, and *c*) they planned to quit/change jobs or to move out of the city over the 1-year course of follow-up. After obtaining informed consent, the interviewer administered a baseline questionnaire, which included information on contraceptive use, reproductive history, socio-demographic characteristics, alcohol use, and environmental and occupational exposures. Beginning from the date of stopping use of contraceptive methods, each woman kept a daily diary to record sexual intercourse, vaginal bleeding, medication, and medical conditions and collected a daily first-morning void urine specimen for hormone assay. Daily diary information and urine specimens were collected for up to 12 months or until a pregnancy was clinically confirmed.

The study was conducted in Anqing Textile Mill during 1997 to 2000. The women were recruited at the local Maternal and Child Health Care Center. Of the total 1,006 newly married women, 35 women were ineligible, 10 women refused, and 961 women were enrolled; 99% of them did not smoke. Hormone assays were performed for 387 women who had provided sufficient diary and urine samples and who did not smoke. A total of 574 women were excluded from the current analysis for the following reasons: 95 women continued to use contraceptives; 121 women declined diary or urine collection; 78 women became pregnant because of contraceptive failure; 53 did not begin recording diaries and collecting daily urine samples immediately after stopping contraception; 8 were lost to follow-up; 7 had menstrual irregularity at baseline; the others did not have adequate diary and urine samples.

### Laboratory assays of urinary PdG, E_1_C, and hCG.

Urine specimens were stored in our field central laboratory at –20°C. Urinary PdG and E_1_C levels were measured by enzyme-based immunoassays (Munro et al. 1991). This method was very sensitive and stable. The minimum detection levels for PdG and E_1_C were 3 ng/mL and 0.096 ng/mL, respectively, and the coefficients of variation measured from the repeated standards were 4.3 and 5.1% respectively. Urinary human chorionic gonadotropin (hCG) levels were analyzed by the immunoradiometric assay (O’Connor et al. 1988). Urine creatinine levels were measured according to the method of Jaffe (Husdan and Rapoport 1968). All PdG, E_1_C, and hCG values were normalized to creatinine values to adjusted for urine concentration. All the urine specimens from each woman were analyzed and tested during a single run and were assayed in duplicates. Discrepancies of more than 3-fold between duplicate assays were presumed to result from technical error, and the assay was repeated. The geometric mean of the replicates has been used to summarize the results for each sample.

### Statistical analysis.

The central focus of our analysis was to examine the independent association between ETS exposure and urinary PdG and E_1_C profiles among eligible menstrual cycles. This required characterization of following key variables: *a*) ETS exposure status; *b*) ovulation status and the day of ovulation in each cycle; *c*) conception status in each ovulatory cycle; and *d*) modeling ETS exposure in relation to menstrual-day–specific urinary PdG and E_1_C levels.

#### ETS exposure status.

As part of the daily diary, information on daily exposure to ETS was obtained. Two specific questions were asked: *a*) Was there anyone who smoked around you at home yesterday? *b*) Was there anyone who smoked around you at your work-place yesterday? If the woman answered “yes” to either of the two questions, we coded the day as having ETS exposure. For a specific cycle, we counted the number of days in the cycle and calculated the percentage of days with ETS exposure. If it was greater than zero, we coded the cycle as having ETS exposure.

#### Day of ovulation.

We used two independent methods to determine the day of ovulation. We first used a previously published E_1_C:PdG ratio algorithm (Baird et al. 1995). Briefly, this algorithm scans 5-day sequences and looks for a 5-day sequence in which the ratio value for the first day is the highest of the five, and the ratio values for each of the last 2 days are ≤40% of the first-day value. The second day in this sequence was designated the day of ovulation or called day of luteal transmission (DLT) for that cycle. This algorithm had several limitations: *a*) The cut-point of a 60% decrease in E_1_C:PdG ratios in the preceding 2 days was derived from previously published data and was subject to the sensitivity of laboratory methods for detecting PdG and E_1_C; *b*) if there were missing data around ovulation, the algorithm may fail to detect the DLT; *c*) multiple DLTs may be detected in one cycle.

To cross-validate the day of ovulation identified by the above method, we also applied a two-piecewise regression model for daily PdG levels to identify the day when PdG started to rise (PdG rising point). This method is based on the fact that PdG remains at a lower level during follicular phase and rises after ovulation. For each cycle, we assumed that log(PdG) values follow a normal distribution with constant mean and variance before ovulation. After ovulation, we modeled the log(PdG) with a normal distribution with a quadratic mean function and constant variance. For the day *j* of cycle *i*, we modeled the log(PdG) value as:


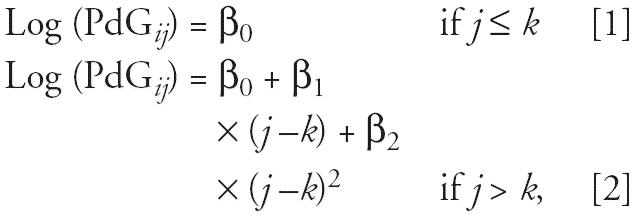


where day *k* + 1 was assigned as the day of ovulation. A “best fit” (maximum *R*^2^) algorithm was applied to identify the turning point *k*. We compared the ovulation day identified by each method. In the subsequent analyses, we only included cycles in which the ovulation days identified by the two different methods were within ± 3 days. To be consistent, we used the ovulation day derived from the PdG rising point in the subsequent analysis.

**Figure 1:**
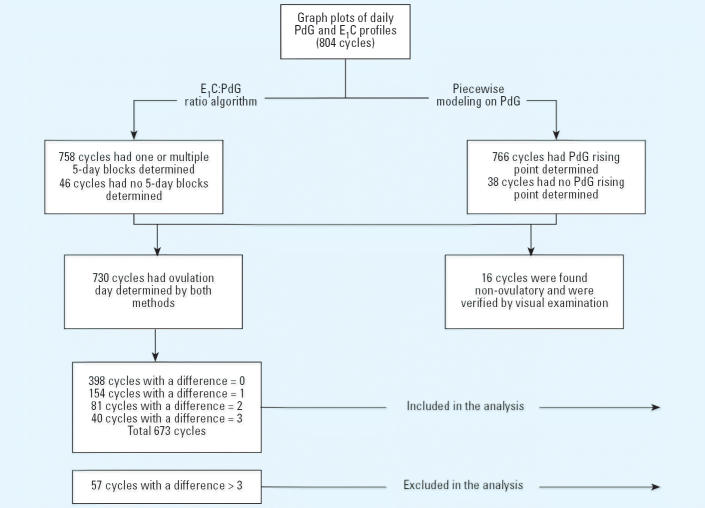
Selection of cycles for inclusion in the analysis.

Figure 1 illustrates the sampling frame and steps we took to determine the day of ovulation. Of the total 1,484 cycles from the 387 women, 804 cycles were selected for urinary hormone analysis. We oversampled the cycles with early pregnancy loss when we selected cycles. Sixteen cycles were found without ovulation by algorithms, and their anovulatory status was verified by graphic examination. After determining the day of ovulation by two different methods, we excluded those cycles for which ovulation could not be determined by one of the methods, or in which comparison of the two methods yielded a difference in ovulation day greater than 3 days. As a result, a total of 673 cycles from 371 women were included in the subsequent analysis. We compared the cycles that were included and excluded and did not find any significant difference in terms of subjects’ demographic characteristics and ETS exposure status (data not shown).

#### Conception status.

A Bayesian model was applied to determine conception status based on daily urinary hCG values. Details can be found elsewhere (Wang et al. 2003). Briefly, urine samples from 37 control women who were not at risk for conception were collected for serving nonconception controls cycles, and urine samples from cycles ending with clinical pregnancy were used for conception control cycles. We assumed that square root of hCG values followed a normal distribution with a constant mean and variance before conception. For postconception, we modeled the square root of hCG with a normal distribution with a quadratic mean function and constant variance, with the mean and variance differing for clinical pregnancy and early pregnancy loss. We chose noninformative proper prior distributions for each parameter and fitted the model using Markov Chain Monte Carlo methods. This model allowed us to calculate a probability of conception for each observed cycle. We defined conception as a probability ≥0.9.

#### ETS exposure and urinary hormone profiles.

Once the ovulation day was identified, we aligned the cycles according to the day of ovulation. The average length of follicular phase was 15.8 days with a range of 6 to 39 days and luteal phase was 15.0 days with a range of 6 to 33 days. We focused on a 20-day window starting from 9 days before ovulation to 10 days after ovulation for comparison of hormone profiles for the following reasons. Most cycles had at least 20 days in length. This ensures comparability of day-specific hormones among cycles, and each cycle has relatively equal contribution to the model parameter estimation. Because the distribution of values for urinary PdG and E_1_C were strongly skewed toward the upper end, we transformed daily urinary PdG and E_1_C to the log scale for analysis. We calculated each day mean log(PdG) and log(E_1_C) for ETS exposure and nonexposure cycles. By inverse transformation, we plotted the daily mean PdG and mean E_1_C by ETS exposure status. We further used linear regression models to examine the associations between ETS exposure and daily log(PdG) and log(E_1_C) levels within the defined window. Because E_1_C and PdG levels fluctuate during preovulatory and postovulatory phase, we adjusted the days relative to ovulation using indicator variables. The basic model was:


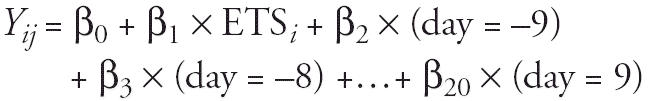


*Y_ij_* was log(PdG) or log(E_1_C) of day *j* in cycle *i*. If a day had missing hormone data, that day was not included in the analysis. Assuming that the missing data were randomly distributed within the window, the model should be valid. The model was also adjusted for potentially important covariates, including age (linear and quadratic terms), body mass index (BMI; linear and quadratic terms), education (high/middle), shift work (yes/no), stress (low, moderate, high), noise exposure (low, middle, high), and dust exposure (low, middle, high). Because there were multiple daily PdG and E_1_C observations from each woman, we applied a generalized estimate equation (GEE) to adjust for intrawoman correlation with SAS procedure GENMOD (SAS Institute, Cary, NC, USA) assuming an exchangeable working correlation structure. We also explored the interaction terms of ETS exposure status and the day indicator variables and found no interactions.

## Results

This is a young, nulliparous cohort. All the women were newly married and were attempting to conceive. Of the 371 women included, none smoke or drink alcohol. They all were full-time textile workers. Table 1 presented the characteristics of women who were included in the analyses stratified by ETS exposure status during the follow-up cycles. Women without ETS exposure were similar to those with ETS exposure in terms of age, height, weight, BMI, education, and occupational exposures. The major occupational exposures in these women were shift work, dust, and noise.

This analysis included 371 women who contributed a total of 1,444 cycles. The average number of cycles followed was 3.8 (range, 1–16). Of the 1,444 cycles, 474 (32.8%) cycles had conception. Of the 474 conceptions, 338 conceptions reached clinical recognized pregnancy; 146 (30.8%) ended with early pregnancy losses. The median time to clinical pregnancy was three cycles.

**Figure 2:**
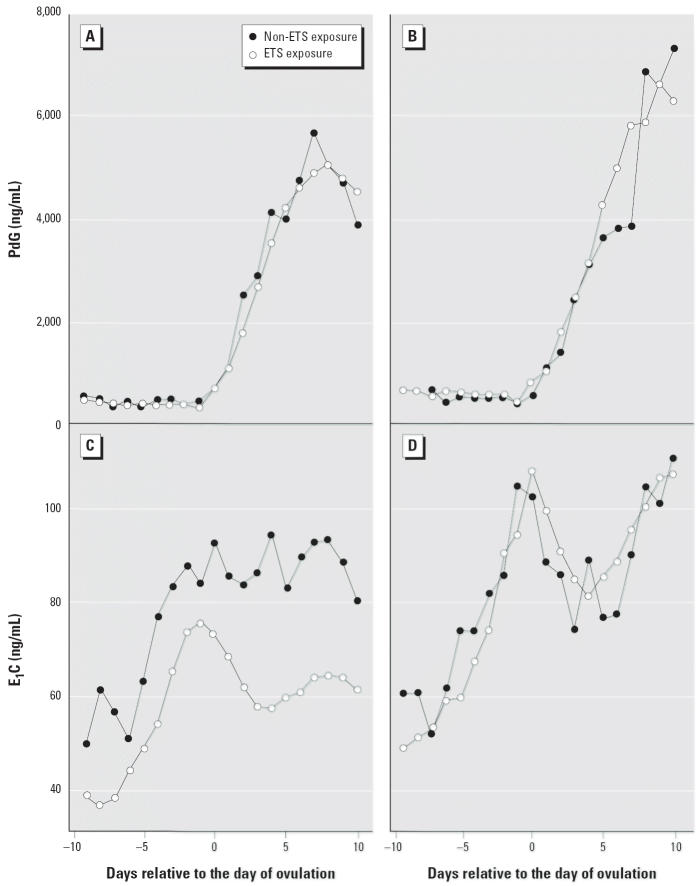
Mean daily PdG (*A*,*B*) and E_1_C (*C,D*) levels in the 20-day window around ovulation by the status of ETS exposure. Of 344 total nonconception cycles (*A*,*C*), 44 had no ETS exposure and 300 had ETS exposure; of 329 total conception cycles (*B*,*D*), 32 had no ETS exposure and 297 had ETS exposure.

Of the 673 cycles included in this study, 344 were nonconception cycles and 329 were conception cycles; 76 cycles did not have ETS exposure and 597 cycles had ETS exposure. Figure 2 illustrates daily mean E_1_C and PdG levels over the 20-day window of the menstrual cycles, stratified by conception versus nonconception cycles. Among the nonconception cycles, ETS-exposed women had a consistently lower daily urinary E_1_C level compared with nonexposed women. Table 2 presents the crude and adjusted associations between ETS exposure and urinary PdG and E_1_C stratified by conception and nonconception cycles. ETS exposure was found to be associated with a lower urinary E_1_C levels (β= –0.43, SE = 0.08, *p* < 0.001 in log scale) among nonconception cycles. Among conception cycles, the association was not significant (β= –0.17, SE = 0.10, *p* = 0.085 in log scale). There was no significant difference in PdG level between nonexposed and exposed women regardless of conception status.

**Figure 3:**
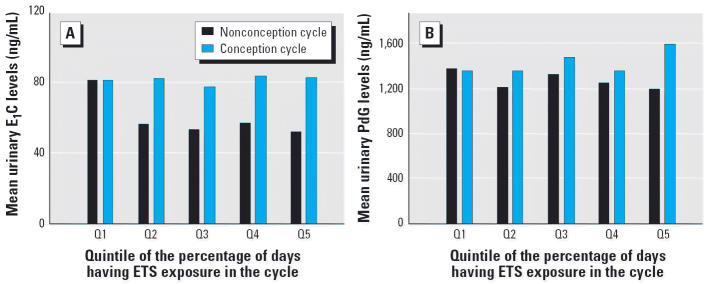
Cycle mean E_1_C (*A*) and PdG (*B*) levels in the 20-day window around ovulation by the quintile of the percentage of days having ETS exposure. Data from 344 nonconception cycles and 329 conception cycles. The ranges of the percentage of days having ETS exposure for each quintile were as follows: Q1, 0–3.3%; Q2, 3.3–18.3%; Q3, 18.4–46.9%; Q4, 47.0–81.0%; Q5, 82.0–100%.

Figure 3 plots the cycle mean urinary PdG and E_1_C levels by the quintile of the percentage of days having ETS exposure for the cycle. The first quintile had higher mean E_1_C than did other quintiles among nonconception cycles, but it did not show a clear dose–response relationship.

## Discussion

The potential adverse reproductive health effects of ETS exposure are of great public health concern even when the reproductive effects of exposure to ETS are of modest magnitude, because ETS exposure is so common and widespread. We recently reported a dose–response relationship between ETS exposure and early fetal loss in the same study cohort (Venners et al. 2004). In this article we reported a significant effect of ETS exposure on urinary E_1_C levels during nonconception cycles but not during conception cycles. The differential effect of ETS on urinary E_1_C levels between conception and nonconception cycles is interesting. One possible explanation is that the E_1_C level was much higher during conception cycles than during nonconception cycles and we did not have enough power to detect the relatively small effect of ETS exposure among conception cycles. This is one of the few prospective studies examining the effect of ETS exposure on urinary hormone markers, and it provides new insight about potential biologic mechanism by which ETS affects reproductive outcomes and stimulates further research in this area. Further studies are needed to understand the interactions between individual susceptibility and ETS exposure on reproductive hormones.

Studying ETS raises unique challenges, including exposure assessment and confounding by active smoking (Seidman and Mashiach 1991). Several limitations need to be taken into account when interpreting the results of this study. All the women subjects were shift workers in a textile industry and were young and nulliparous. Therefore, caution is needed before generalizing our findings to other populations. We did not have biochemical markers of ETS exposures, and we relied only on women’s self-reporting of both active and passive cigarette smoking. The sample size of non-ETS group was relatively small. The ovulation days were estimated from urinary PdG and E_1_C levels, and we included only the cycles in which the ovulation days identified by two methods were within ± 3 days, and this may be still subject to errors. To address this issue, we conducted sensitivity analysis by changing the inclusion criterion of the cycles in which the ovulation days identified by two methods were the same, within ± 1 day, within ± 2 days, within ± 4 or more days, and repeated the analysis respectively. The results did not change significantly (data not shown). Furthermore, ETS exposure at different days of the menstrual cycle may have different effects, and we did not have adequate sample size to examine the potential timing effect of ETS exposure.

Our study also has the following strengths: The accurate determination of day of ovulation is critical for appropriate comparisons of hormone levels among individuals, given that hormone levels change significantly over a menstrual cycle. In this study, the day of ovulation for each menstrual cycle was cross-validated by two methods. This allowed us to align the individual menstrual cycles according to ovulation day. This was a prospective study, and the ETS exposure status was based on daily diary recording during the menstrual cycle, which eliminated potential recall biases commonly encountered in retrospective studies. In many previous studies, it was difficult to tease out the effect of active smoking versus ETS exposure when the study population contained a high proportion of women who were exposed to both active and passive smoking. In this study, all the women were nonsmokers. In addition, these women were homogeneous in terms of sociodemographic characteristics and occupation; thus, this study is less likely confounded by other environmental exposures.

The observed association between ETS exposure and urinary E_1_C level is biologically plausible. Cigarette smoking had been suggested to have antiestrogenic effects (Baron et al. 1990; Spangler 1999). Evidence showed that smoking reduces the risk of endometrial cancer, increases the risk of osteoporosis, and decreases the age of menopause. Our finding is also consistent with a number of studies indicating the effects of active smoking on estrogen levels. MacMahon et al. (1982) reported lower urinary levels of estrone, estradiol, and estriol during the luteal phase of the cycle among the premenopausal smokers compared with non-smokers and ex-smokers and suggested that smoking might reduce luteal estrogen production. Subsequently, Mochizuki et al. (1984) reported significantly lower estrogen levels in pregnant smokers compared with pregnant nonsmokers. Basu et al. (1992) reported that smoking and oral contraceptives independently lower serum estradiol and progesterone concentrations in premenopausal women. Westhoff et al. (1996) further reported that cigarette smoking was associated with decreased mid-cycle and lutealphase estradiol levels.

Other studies of smoking and reproductive hormones yielded negative results. Friedman et al. (1987), in a study of 9 postmenopausal smokers and 16 nonsmokers, found that estrone, estradiol, dihydrotestosterone, and dehydroepiandrosterone sulfate (DHEA-S) did not differ between the two groups. Key et al. (1991) further compared serum concentrations of estradiol, progesterone, and DHEA-S, and urinary excretion rates of six steroids of predominantly adrenal origin, in a large cohort of healthy premenopausal and postmenopausal female smokers and nonsmokers, and found that cigarette smoking does not affect serum estradiol. Thomas et al. (1993) from a study of 25 premenopausal cigarette smokers and 21 nonsmokers also reported no significant difference in urinary concentrations of estradiol, estrone, or estriol.

The possible explanations for these discrepancies include inadequate sample size for the negative studies, population differences, and, most important, lack of control of the timing of ovulation relative to the hormone sampling. As is well known and also seen from our data, reproductive hormones fluctuate over a menstrual cycle, and the timing of the specimen collection is likely to be a critical factor in making comparisons among groups or studies. It is conceivable that without controlling for ovulation, it would be difficult to make valid comparison in hormone levels between exposed and unexposed individuals. The selection of serum versus urinary hormone markers and laboratory methods may also account for some differences. In addition, reproductive hormones are affected by both physiologic and pathologic states, including a woman’s age, BMI, pre- versus postmenopause, ovulation versus nonovulation cycle, conception versus nonconception cycle, use of oral contraceptives, or other exogenous sex hormones.

In summary, this prospective study indicated that ETS exposure in demographically homogeneous nonsmoking women was associated with significantly decreased urinary E_1_C levels throughout the nonconception menstrual cycles, suggesting that the adverse reproductive effect of ETS may act in part through its antiestrogen effects.

## Figures and Tables

**Table 1:** Characteristics of the 371 newly married young women by status of any ETS exposure, Anqing, China (mean ± SD or %).

Characteristic	Nonexposed (*n* = 25)	ETS exposed (*n* = 346)	*p*-Value
Age (years)	25.1 ± 1.8	24.9 ± 1.6	0.450*^a^*
Height (m)	1.6 ± 0.1	1.6 ± 0.1	0.481*^a^*
Weight (kg)	48.1 ± 6.3	49.3 ± 5.8	0.320*^a^*
BMI (kg/m^2^)	19.6 ± 2.1	19.8 ± 2.1	0.565*^a^*
Age at menarche (years)	14.5 ± 1.5	14.7 ± 1.4	0.562*^a^*
Cycle length (days)	30.0 ± 7.3	30.8 ± 5.4	0.547*^a^*
Follicular phase (days)	13.8 ± 6.6	15.9 ± 5.4	0.139*^a^*
Luteal phase (days)	16.2 ± 3.9	15.0 ± 3.1	0.118*^a^*
Shift work	96.0	95.7	0.936*^b^*
Education			0.177*^b^*
Middle school	60.0	63.1	
≥High school	40.0	37.9	
Dust exposure			0.489*^b^*
Low	28.0	33.9	
Moderate	28.0	36.8	
Heavy	44.0	29.3	
Noise exposure			0.665*^b^*
Low	24.0	26.6	
Moderate	36.0	35.7	
Heavy	40.0	37.7	
Perceived stress			0.224*^b^*
Low	68.0	63.2	
Moderate or high	32.0	36.8	
Previous contraception			0.290*^b^*
Condom	43.5	29.8	
Intrauterine device	0	4.0	
Pill	0	0.9	
Others	21.7	15.5	
No contraception	34.8	49.8	
ETS exposure			
At home only		70.8	
At work only		2.0	
Both at work and at home		27.2	

a *t*-Test.

b Chi-square test.

**Table 2:** Associations between ETS and daily urinary PdG and E_1_C levels.

	Crude			Adjusted		
Levels (pg/mL)	β	SE	*p*-Value	β	SE	*p*-Value
Nonconception cycles						
Daily urinary log(E_1_C)	–0.44	0.08	< 0.001	–0.43	0.08	< 0.001
Daily urinary log(PdG)	–0.08	0.15	0.600	–0.07	0.15	0.637
Conception cycles						
Daily urinary log(E_1_C)	–0.14	0.09	0.09	–0.17	0.10	0.085
Daily urinary log(PdG)	–0.07	0.14	0.654	–0.10	0.12	0.408

Sample size: nonconception cycles, 6,880 days (344 cycles); conception cycles, 6,580 days (329 cycles). Twenty indicator variables were created to represent each day in the 20-day window and were put in the adjusted models along with other covariates: age, age squared, BMI, BMI squared, education (high/middle), shift work (yes/no), stress (low, moderate, high), noise exposure (low, middle, high), and dust exposure (low, middle, high). All models used the GEE method to adjust for intrawoman correlation in cycles. Among the other covariates, shift work was found to be associated with a lower log(E_1_C) levels (β= –0.25, SE = 0.12, *p* = 0.025); all other covariates were nonsignificant.
